# Editorial: Psychocardiology in socially disadvantaged groups

**DOI:** 10.3389/fpsyt.2025.1704464

**Published:** 2025-11-03

**Authors:** Don Byrne, Marlise E. Alvarenga, Kai G. Kahl

**Affiliations:** ^1^ Australian National University, Canberra, ACT, Australia; ^2^ Federation University Australia Institute of Health and Wellbeing, Ballarat, VIC, Australia; ^3^ Medizinische Hochschule Hannover, Hanover, Germany

**Keywords:** psychocardiology, CVD risk, social disadvantage, depression, epidemiology

At first glance, the construct of social disadvantage (SD) appears to be a simple one. From a global perspective, authoritative bodies such as the World Health Organization (WHO) and the World Bank (1) definitively equated the pathogenic effects of social disadvantage with poverty, and went on to exhaustively document its deleterious impact on health at the population level. Kennedy (2) aptly summarised this as the “plagues of poverty”. Along the way, it has been common to frame SD under the broader umbrella of social determinants of health (SDOH). However they are viewed and conceptualised, these determinants - the conditions under which people are born, grow up, live, work, and age - are indisputably accepted as fundamental and central drivers of both communicable and non-communicable diseases.

As convincing and undeniable as this macro-social perspective is, unpacking SDH in more detail reveals a layered and dynamic construct. Social disadvantage, as one, albeit multi-faceted, manifestation of SDH, rarely exists as an absolute entity but rather as a multitude of potential drivers of inequality (as we will see a little further on in this narrative), cascading, interacting and accumulating across the lifespan. Thus, when looking at SD within the SDH framework, we are faced with a complex constellation of interacting social, economic, and environmental forces that collectively drive inequalities in health and healthcare. In particular, in relation to the intent of this Research Topic, we consider the overall impact of SDH on the development, course, and outcomes of cardiovascular disease (CVD). From the global perspective of psychocardiology, it is now clear that low socio-economic status endows a documented risk of premature coronary heart disease in adults (3).

In this light, then, a plethora of factors, conditions and circumstances have been revealed in a substantial corpus of published works to identify aspects of the social environment that characterise states of disadvantage (4). In a recent examination of social inequalities in relation to CVD, the Australian Institute of Health and Welfare (5), drawing on the formative work of Wilkinson and Marmot (6), lists a range of social factors that contribute statistically to CVD risk, including socioeconomic position, early life experiences, social exclusion, lack of social capital, challenging employment and working conditions, and inadequate or unavailable housing. A recent Oxford Research Briefing (7) adds low income and poverty, discrimination (racial, ethnic, and sexual), and gender to the potential drivers of SD. The review goes on to suggest that manifest SD may impose barriers on such essentials as education, healthcare and employment. Moreover, as previously suggested, domains of disadvantage are unlikely to exist as absolutes but may be seen as statistical cascades and gradients, accumulating over time and impacting the ultimate risk of CVD (4, 8).

Thus, while socioeconomic disadvantage – poverty, as Kennedy (2) sees it in its most extreme form – offers a strong starting point for considering aspects of the social environment as constituting health risks, it is now clear that socioeconomic disadvantage alone is too simplistic a metric with which to account for how SD, as a more refined construct, may confer an elevated risk of CVD.

At a population level, the Australian Institute of Health and Welfare (AIHW) published a major report in 2019, which drew on census data from the entire Australian population to persuasively argue that socioeconomic disadvantage (so-called, since the overall metric also included measures of education and of housing suitability and tenure, along with income alone) had a significant negative effect on both CVD morbidity and mortality, and in both men and women. In light of the brief discussion presented above, three questions have immediately begged our attention.

First, we asked, can SD, broadly defined, influence the risk of CVD through the intervening effect of already historically identified risk factors (RFs) for CVD? Here, the recent evidence is voluminous. SD has been individually linked to diabetes (9, 10), dyslipidaemia (11), obesity (12, 13), and smoking (14), such that manifest SD is statistically coupled with elevated levels of CVD RFs. SD has also been related through multivariate analyses to a collective basket of historical RFs, including arterial hypertension, Body Mass Index (BMI) and obesity, dyslipidaemia, and smoking (15). It appears as undeniable, then, that the reported links between SD and CVD may arise through the intervening influence of one or more of a set of the historically identified RFs for CVD.

Second, we asked, can SD create a social environment which, in turn, elevates the risk of CVD? There is good evidence (16) that SD is closely linked to social isolation and loneliness, and that this psychosocial condition elevates the risk of CVD (17). We see this as another plausible hypothesis for the link between SD and CVD.

Third, we asked, could SD influence the risk of CVD through the intervening effects of emotional distress? Recent studies (18) now support the view that SD increases the risk of depression, which in turn is now undeniably linked to a high risk of CVD (19, 20). Once more, a credible (albeit indirect) link between SD and CVD is clearly indicated.

Of course, it is well beyond the scope of this Editorial to examine all the potential drivers of disadvantage in relation to CVD risk in full. The corpus of existing literature is enormous, and it would take a volume of significant size – and perhaps more – to achieve this. Rather, it is the intention of this brief narrative to underscore the true complexity of what, on the surface, might appear to be a simple link between SD and CVD. In doing so, we have aimed to provide a suitable context for the original works which have been presented for publication in this Research Topic of *Frontiers in Psychiatry*.

Recognising the complex bio-psycho-social network that associates SD with CVD, five original articles have been published in the Research Topic. Schaefer et al. demonstrated that individuals with Borderline Personality Disorder (BPD) are inordinately prone to elevated epicardial adiposity and, through this, to a potentially elevated risk of CVD. In light of a historically established association between BPD and SD, the implicit link to SD and CVD risk is clear. Kennedy persuasively argued that SD is linked to sleep insufficiency and, through this potential mechanism, to an elevated risk of CVD. Friedman et al. reported associations between obesity and distress/depression, and thence, hypothetically, to an elevated risk of CVD. As we have noted earlier in this editorial, obesity also has a historically established association with SD, thus drawing SD into play in this complex network of CVD risk. Kirchberger et al. showed that a significant number of survivors of incident CVD experience distress and depression during the 5-year post-incident period, and many of these patients exhibit poor mental health literacy (MHL). Among them, the elderly and those with little education– both characteristics of SD – have the poorest MHL. It would be interesting to see whether these individuals went on to experience elevated levels of repeat incident CVD. Finally, Accinni et al. informed us that individuals suffering from supraventricular tachyarrhythmias (STs) not infrequently suffer from comorbid demoralisation. Demoralisation is also associated with SD and, via postulated links with depression, may also presage an elevated risk of CVD.

It is with pleasure, therefore, that we can recommend each of these persuasive new studies as further evidence of the now emerging, albeit complex, association between SD and CVD.
